# Sleep apps: current limitations and challenges

**DOI:** 10.5935/1984-0063.20200036

**Published:** 2021

**Authors:** Sachin Ananth

**Affiliations:** West Hertfordshire Hospitals NHS Trust, Department of Respiratory Medicine - Watford - United Kingdom.

**Keywords:** Mobile Applications, Validation Study, Smartphone, Polysomnography, Sleep

## Abstract

Sleep app ownership is increasing exponentially, due to their accessibility and ease-of-use. However, there are several concerns regarding the use of sleep apps. Few sleep apps demonstrate empirical evidence to support their claims, and if they do, this evidence can be based on signiﬁcant methodological limitations. In addition, there are data privacy concerns with regards to sleep apps, which share sensitive user data with business and marketing partners, unbeknownst to their users. Moreover, sleep apps may increase engagement with healthcare professionals, which may place additional strain on under-pressure sleep services. This would be compounded by the fact that some sleep apps produce many false positives, and clinicians would need more time to analyze the data provided by these apps. In the future, sleep apps must undergo rigorous validation studies and grant more autonomy to their users over how their data is shared.

## INTRODUCTION

A sleep app is defined as a specialised programme downloaded onto medical devices, marketed with the assertion that it performs sleep monitoring or sleep-related interventions^1^. Sleep app ownership is increasing exponentially, due to their affordability, ease-of-use and the ubiquitous nature of smartphones; there will be an estimated 6 billion smartphones in use by the end of 2020^2^. Sleep apps are used to screen for various conditions, including obstructive sleep apnoea, insomnia and periodic limb movement disorder^3-5^. While sleep apps have the potential to raise awareness of sleep conditions and promote healthy sleep habits, there are several concerns regarding their use.

This article aims to outline the current limitations of sleep apps and challenges which need to be overcome before sleep apps can be safely integrated into clinical practice. In particular, many sleep apps are not supported by high-quality evidence. Moreover, the sensitive data collected by sleep apps may be breached and exploited by the apps’ developers. Finally, sleep apps may place added strain on sleep services.

### Lack of evidence-based medicine in sleep apps

Many sleep apps are not supported with evidence-based medicine. 32.9% of sleep apps available in the Google Play Store demonstrated empirical evidence to support their claims, and 15.8% were developed with the input of clinicians^6^. To our knowledge, there are no similar studies evaluating sleep apps in other app stores (such as Apple’s App Store). Furthermore, a recent systematic review of sleep apps which detect sleep parameters showed that only 3 out of 73 apps had undergone validation studies using the gold standard: polysomnography^7^. In these validation studies, each subject underwent polysomnography while simultaneously using the sleep app. All 3 validation studies demonstrated weak correlation between polysomnography and the sleep apps, including the detection of sleep stages (Table 1)^8-10^. This is particularly relevant as all 3 apps claim to have a “smart alarm” that wakes up users during non-REM sleep. Moreover, several “smart alarms” assume that sleep cycles are 90 minutes long. This is opposed by a landmark study, which showed that sleep cycle durations vary from 41.3 to 97.6 minutes^11^. There is even conflicting evidence regarding the relevance of sleep stage awakening on the duration and severity of sleep inertia^12^, which undermines the basis of “smart alarms”. The lack of rigorous evidence in sleep apps is perpetuated by their frequent classification as “entertainment” products, with fine print absolving them of medical responsibility^13^. More validation studies of sleep app use in healthy adults are needed, as most of the current evidence is based on children with suspected or confirmed sleep disorders^8-9^.

**Table 1:** Results of validations studies of sleep apps which claim to detect sleep parameters. All comparisons are made with polysomnography^8-10^.

Sleep App	Study Population	Exclusion Criteria	Study Findings
Sleep Cycle^8^	- n=25 (22 suspected OSA, 3 healthy volunteers); - Age (years) = 8.0 ± 3.6; - % male = 56.	- Complex genetic or craniofacial disorders.	- No correlation with polysomnography in the measurement of total sleep time (CCC 0.22, *p*=0.36); - No correlation in the measurement of sleep latency (CCC 0.05, *p*=0.16); - No correlation in the detection of sleep cycle stages (data not provided).
MotionX 24/7^9^	- n=78 (all suspected OSA); - Age (years) = 8.4 ± 4.0; - % male = 65.	- Conditions affecting motor control or limb movement.	- Over-estimated total sleep time by 106 minutes (*p*<0.0001); - Over-estimated sleep efficiency by 17% (*p*<0.0001); - Over-estimated sleep period time by 16 minutes (*p*<0.0001).
Sleep Time^10^	- n=20 (all healthy volunteers); - Age (years) = 39.5 ± 12.4; - % male = 60.	- Diagnosed sleep disorder.	- No correlation with polysomnography in the measurement of sleep efficiency (*p*=0.59) or sleep latency (*p*=0.09); - Under-estimated light sleep by 27.9% (*p*<0.0001); - Over-estimated deep sleep by 11.1% (*p*<0.0001).

Age expressed as mean ± standard deviation; OSA: Obstructive sleep apnoea; CCC: Concordance correlation co-efficient.

Furthermore, sleep apps may rely on evidence with significant methodological limitations. For example, the “Pzizz” app, which is promoted on the UK’s National Health Service website, is supported by a single clinical trial^14^. 16 participants used “Pzizz” during 20-minute naps every day for 2 weeks. This resulted in increased well-being survey scores at the end of the study period. However, the study lacked a control group and so it is unclear whether the act of daytime napping was the cause of the increased well-being scores. Furthermore, the well-being survey was created using “Pzizz promotional material”^14^, rather than a validated survey. In addition, most studies of sleep apps used to detect obstructive sleep apnoea are undertaken in a controlled environment, rather than at the users’ homes, which is where these apps will be primarily used^15^. This reduces the external validity of these studies, and may explain why some sleep apps have weak diagnostic sensitivity for obstructive sleep apnoea (as they are unable to detect snoring in a noisier, less controlled environment)^16^. Thus, it is important to critically appraise any evidence supporting sleep apps.

### Data privacy concerns

There are data privacy concerns with regards to sleep apps. The privacy notice of Sleep Number states that user data “may be shared” with business and marketing partners for the purpose of “research, analysis or administering surveys”^17^. Another app (Sleep Cycle) collects data about sleep habits worldwide, with its privacy policy stating that users consent to give the app access to their location if they use the app^18^. However, one can question the ethicality of placing this information in a privacy policy, since 91% of app users accept legal terms and conditions without reading them^19^. Therefore, it is unclear whether the users of sleep apps are aware that sensitive data is shared.

These examples of data sharing act as a microcosm of the data privacy concerns surrounding health apps. A systematic review of 24 health apps found that user data was shared with 46 “third parties” and 216 “fourth parties”^20^. Another review of 20 popular health apps showed that most violated the EU’s General Data Protection Regulation, so that de-anonymized personal details (such as locations) could conceivably be shared with third parties^21^. Sleep app data is vulnerable to these breaches, as this data is not protected by the privacy laws that apply to data collected in sleep clinics^22^. Sharing of de-anonymized health app data opposes the beliefs of health app users: 67% of users state that anonymity is “very” or “extremely” important^23^.

These data breaches are particularly worrying with regards to sleep apps, as they collect highly sensitive information, such as sleep habits. One data privacy expert has even stated that third parties may be able to “interpret raw data” collected by sleep apps to identify when users engage in sexual activity^22^. This data may be used to develop targeted advertising, whereby online advertisements are tailored to an individual based on data provided by their sleep app^24^. This is already common practice when mobile health data is shared with third parties: 66% of these third parties collected the data for advertising and analytics^20^. For example, an expert in internet law has hypothesized that the data provided by a user’s sleep app may suggest that the user engages frequently in sexual intercourse, leading to marketing companies sending multiple online advertisements for contraceptives^25^. Other people using the same Internet device may see these online advertisements, and so this may lead to intrusions into a sleep app user’s privacy. Furthermore, online hackers are increasingly able to de-anonymize data shared by health apps; the names and fitness records of military personnel working in a high-security nuclear base were accessed using data provided by the fitness app Strava^26^. Similar data breaches are possible with sleep apps. For instance, some sleep apps store unencrypted audio files of users’ sleep sounds. Malicious apps are able to access these sleep recordings if they have read storage permission and can then send these recordings to external servers if they have internet permission^27^.

### Impact on clinical practice

Sleep apps will likely increase engagement with healthcare professionals^28^, but this may have several unintended consequences. In the UK, the increase in sleep clinic referrals will put more strain on sleep services, which have already seen the number of sleep tests double from 2007-08 to 2016-17^29^. This added strain may be unnecessary since some sleep apps produce many false positives, such as an app that detects snoring^30^. Furthermore, clinicians may require additional training and time to analyze data from sleep apps, as this data can be presented in many different ways (such as values, graphs or a combination of both)^1^. This is because there are no clear standards in the way that sleep app data should be presented^31^. Also, clinicians may struggle to interpret the “sleep scores” provided by sleep apps, since the algorithms used to formulate these scores are often not disclosed, and neither are the normal ranges^32^. This issue is compounded by the fact that few sleep apps allow users to export their data for further analysis, and so clinicians may not be able to access the raw data that contributes to the “sleep scores”^7^. Moreover, it will be difficult to integrate sleep app data into patient health records, as many countries lack fully-digitized patient records; for example, only 12% of hospital trusts in the UK are fully-digitized^33^. Finally, sleep apps may even damage the doctor-patient relationship, as they could provide conflicting opinions to users’ doctors^34^.

### Recommendations

While sleep apps now appear to be a mainstay of sleep self-management, more work is needed for them to be used with confidence. Sleep apps must undergo rigorous validation studies to ensure that their claims are evidence-based. These validation studies should compare the sleep data generated by the app with sleep data from a relevant gold-standard (such as polysomnography). The sharing of user data with third parties must be fully disclosed in a more transparent manner than fine print in privacy notices, and users should retain autonomy over how their data is shared. Finally, the developers of sleep apps should listen to feedback from clinicians, such as suggestions on how to improve usability, to facilitate the smooth transition of these apps into clinical practice.
